# Editorial: Harnessing Omic Sciences to Unravel Mechanisms and Therapeutic Targets in Allergic Diseases

**DOI:** 10.3389/fimmu.2026.1832049

**Published:** 2026-04-16

**Authors:** Rubén Fernández-Santamaría, Urszula Radzikowska, Blanca Cárdaba, José Antonio Cañas

**Affiliations:** 1 Immunology Department, Health Research Institute-Fundación Jiménez Díaz, Universidad Autónoma de Madrid (IIS-FJD, UAM), Madrid, Spain; 2 Swiss Institute of Allergy and Asthma Research (SIAF), University of Zurich, Davos, Switzerland; 3 Centro de Investigación Biomédica en Red (CIBER) de Enfermedades Respiratorias (CIBERES), Madrid, Spain

**Keywords:** allergy, biomarker, mechanism, omics, therapeutic strategies

Over the last decades, advances in omics technologies have substantially expanded our understanding of the immunological processes underlying allergic diseases, redefining how disease endotypes are established. These high-throughput approaches have important clinical implications, enabling the discovery and screening of novel diagnostic biomarkers and the identification of potential therapeutic targets. Omics disciplines encompass multiple molecular layers, including genomics and epigenomics (DNA and small RNA levels), transcriptomics (RNA level), proteomics (protein level), and metabolomics and lipidomics (metabolite level).

Nevertheless, the vast amount and complexity of the data generated require sophisticated bioinformatic tools and integrative analytical strategies. Importantly, the integration of multi-omics data with non-omics information, such as clinical and laboratory parameters, remains a significant challenge in this field.

Artificial intelligence (AI), particularly machine learning (ML), offers powerful approaches for data integration and uncovering how distinct biomarkers are interconnected through immune mechanisms. These strategies allow us to move beyond single-disease analyses to identify shared molecular signatures across related diseases, such as allergic rhinitis and atopic dermatitis. In this context, Zhang et al. identified 36 differentially expressed genes common to both diseases that are mainly associated with epithelial barrier dysfunction and immune activation pathways. Using ML algorithms, five potential biomarkers (*CD274*, *SERPINB4*, *CYP2E1*, *SPRR1B*, and *FOLH1*) were isolated, underscoring the interplay between innate and adaptive immunity and epithelial-immune cell interactions (Zhang et al.).

Epigenomics, which is mainly focused on post-transcriptional gene regulation mechanisms, has emerged as a useful tool for identifying disease-specific patterns. In this context, genome-wide DNA methylation profiling in children with cow’s milk allergy (CMA) revealed differential methylation patterns (DMPs) in specific CpG islands, particularly between IgE-mediated CMA, non-IgE-mediated CMA, and non-allergic children. These DMPs were associated with genes such as *LDHC*, *TRAF3IP3*, *TMCO3*, and *BCL11* (Lopez-Gomez et al.). Interestingly, differential CpG methylation was also detected in genes potentially linked to tolerance acquisition, distinguishing tolerant from non-tolerant children after a 6-month exclusion diet (e.g., *RNF39*, *LTB4R*, or *LTB4R2*). Overall, these findings are relevant for the identification of diagnostic and prognostic biomarkers in food allergies and tolerance development.

In addition to host genetics and epigenetics, allergy susceptibility is also influenced by microbiome composition. In a study involving different murine strains (BALB/c and C57BL/6), maintained under germ-free or specific-pathogen-free conditions, Hornikova et al. demonstrated that strain- and microbiota-dependent differences in type 2 (T2) immune responses following epicutaneous allergen sensitization and challenge (Hornikova et al.). Microbiome sequencing revealed associations with differential MCPT-1 production in the jejunum and serum, along with increased *Alox5* mRNA expression. These findings support the concept that both the host genome and microbiome composition shape food allergy development and severity.

Cellular metabolism and its derived metabolites also play a key role in allergic diseases. Lipidomics provides detailed insight into bioactive lipid mediators; however, technical limitations, such as low cell numbers and background noise, pose challenges. To address this, Delgado-Dolset et al. developed a novel correlation-based workflow that links increased cell numbers with lipid detection, enabling the reliable identification of cell-derived lipids (Delgado-Dolset et al.). Validation experiments using human lung microvascular endothelial cells (HMVEC-d) stimulated with sera from acute and baseline anaphylactic patients revealed significant alterations in lipid composition, particularly in sphingolipids, highlighting their potential as predictive biomarkers and indicators of disease dynamics.

From a therapeutic perspective, computational tools can also contribute to improving strategies against IgE-mediated allergic diseases. B-cell epitope prediction (BCEP) represents a promising approach to anticipate vaccine immunogenicity and immune recognition (Falcon et al.). Understanding how IgE and IgG antibodies recognize allergen epitopes is essential for designing effective immunotherapies. In particular, focusing on discontinuous (conformational) B-cell epitopes may better reflect physiological antibody recognition. The inclusion of specific B-cell epitopes in vaccine constructs could induce blocking IgG antibodies, thereby reducing IgE-mediated effector responses. Thus, computational BCE prediction tools could guide the rational design of safer and more effective allergy vaccines.

**Figure 1:**
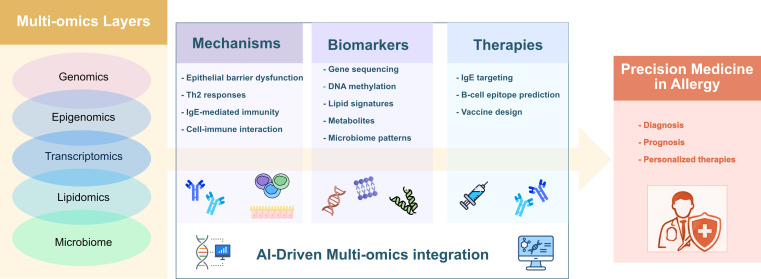
Multi-omics in precision medicine in Allergy. The integration of multi-omics data improves our understanding of immune mechanisms, facilitates biomarker identification, and supports the development of targeted therapies. Digital tools and artificial intelligence (AI) are essential for integrating these complex datasets and translating them into clinical practice within the framework of precision medicine in allergy.

In summary, the studies published in this Research Topic illustrate how omics technologies are transforming our understanding of allergic diseases, from molecular mechanisms to biomarker discovery and therapeutic innovation (**Figure 1**). Nevertheless, further progress in multi-omics integration, supported by digital and AI-driven tools, will be essential to translate these findings into precision medicine approaches. Improved analytical workflows will help distinguish biologically meaningful signals from background noise, ultimately enhancing the diagnosis, prognosis, and management of allergic diseases and contributing to more efficient healthcare systems.

